# Supplementary material for: Formation and Stability of Pea Proteins Nanoparticles Using Ethanol-Induced Desolvation
Source: Nanomaterials (Basel). 2019 Jun 29;9(7):949. doi: 10.3390/nano9070949 (PMC6669580; doi:10.3390/nano9070949)
Supplement: Supplementary file 1 [file nanomaterials-09-00949-s001.pdf]

Supplementary data

Formation and Stability of Pea Proteins Nanoparticles Using Ethanol-Induced Desolvation

Chi Diem Doan [1,*] and Supratim Ghosh [1,*]

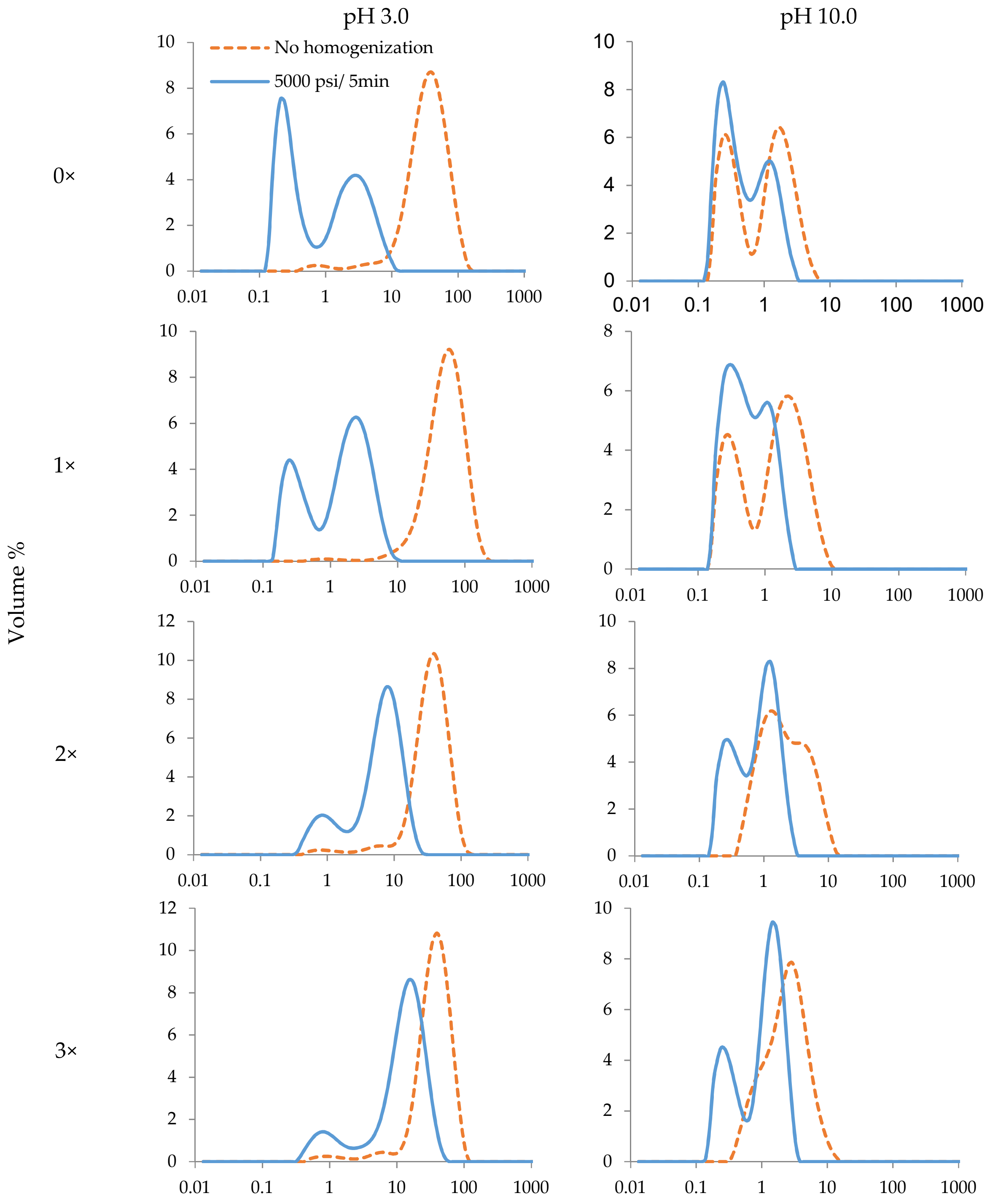

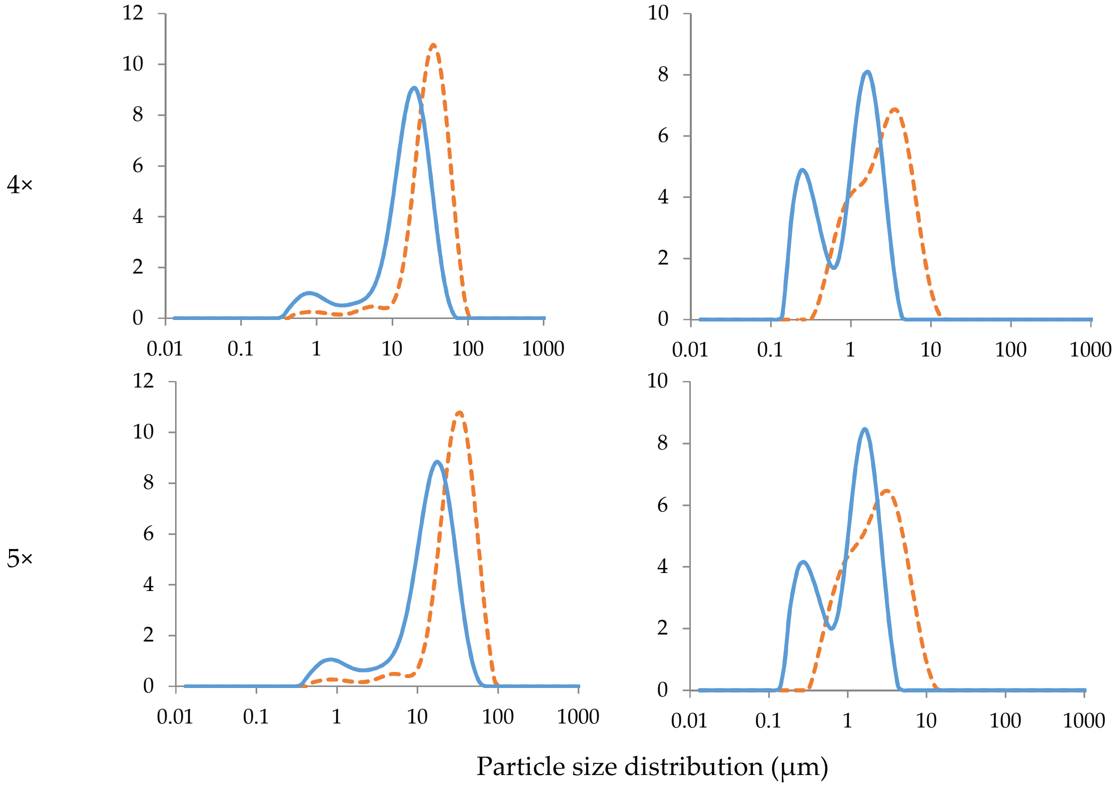

Figure S1. Particle size distribution of desolvated pea protein particles at pH 3 and pH 10 before and after homogenization at 5000 psi for 5 min.

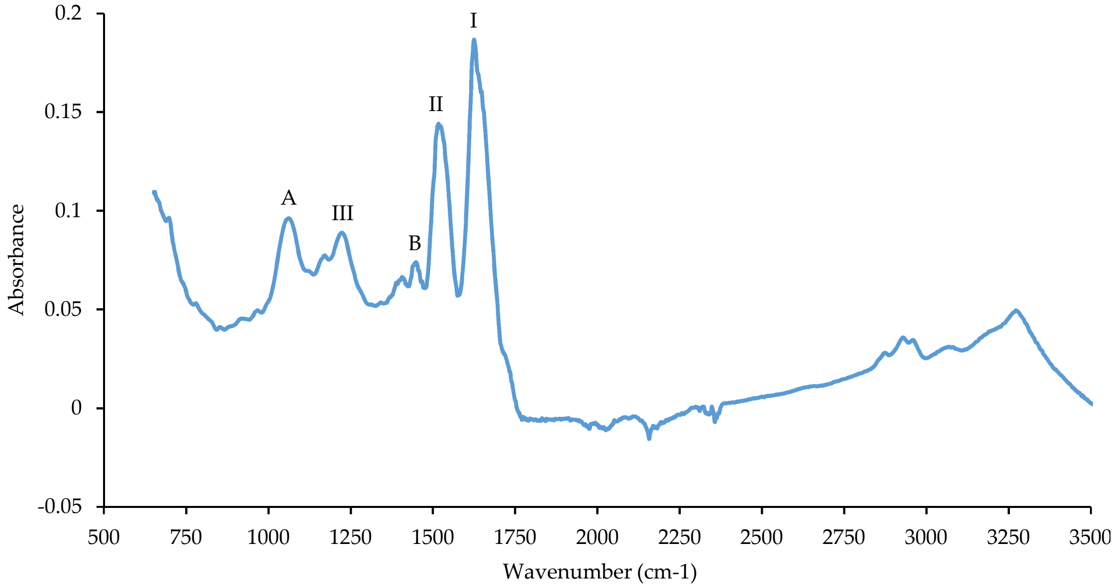

Figure S2. A representative FTIR spectra of 3X desolvated PPN and at pH 3 synthesized at 25 °C.

Band A: band around 1100 cm^{-1}, C–O and C–C stretching, NH stretching
Amide III: band around 1240 cm^{-1}, C–N stretching, NH bending
Band B: band around 1420 cm^{-2}, C-H bending, mainly originated from the deformational vibrations of the CH2 functional group
Amide II: bands around 1480 and 1575 cm^{-1}, NH bending, CN stretching
Amide I: bands around 1600 and 1690 cm^{-1}, C=O stretching

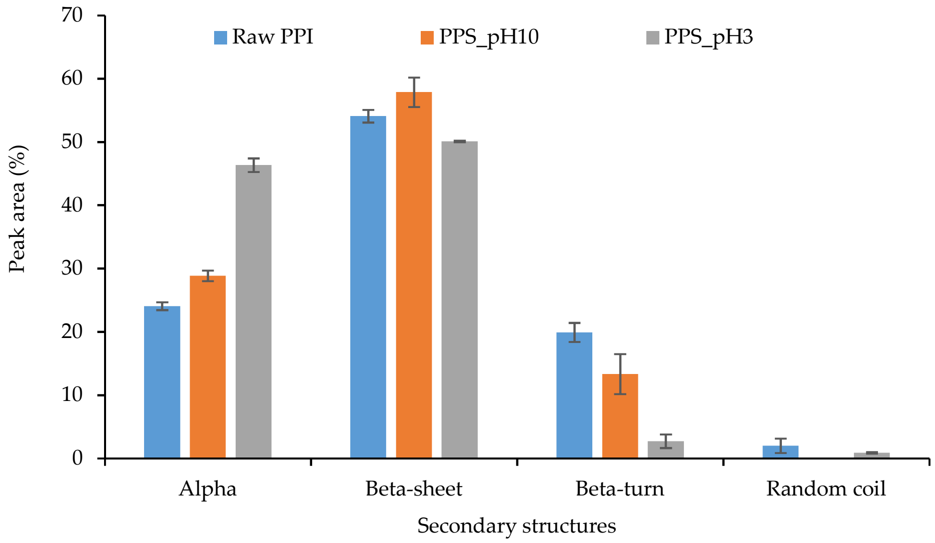

Figure S3. Secondary structure components of raw pea protein isolates (PPI), supernatant soluble pea protein at pH 10 and pH 3 without desolvation.

2X – pH 7 3X – pH 7

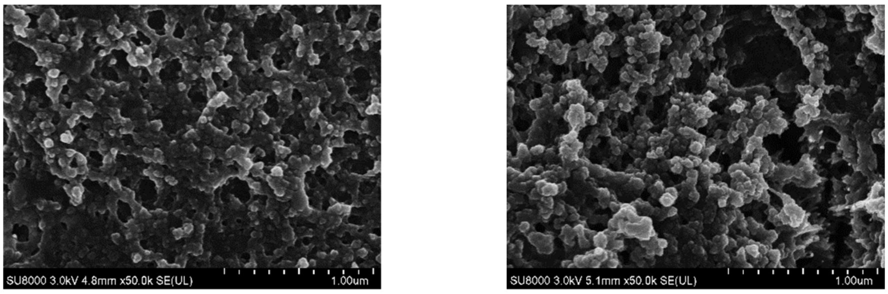

Figure S4. SEM images of re-dispersed pea protein particles at pH 7.
